# 

**Published:** 1867-08

**Authors:** 


### Books and Pamphlets Received.

The Physiology and Pathology of the Mind. By Henry Maudsley, M. D., London, Physician to the West London Hospital, Honorable Member of the Medico-Psychological Society of Paris, formerly Resident Physician to the Manchester Royal Lunatic Hospital, etc. New York: D. Appleton & Co. Buffalo: Breed, Lent & Co.

A Treatise on Human Physiology, designed for the use of Students and Practitioners of Medicine. By John C. Dalton, M. D., Professor of Physiology and Microscopic Anatomy in the College of Physicians and Surgeons, New York; member of the New York Academy of Medicine; of the New York Pathological Society; of the American Academy of Arts and Sciences, Boston, Mass., etc. Fourth edition, revised and enlarged, with two hundred and seventy-four illustrations. Philadelphia: Henry C. Lea, 1867. Buffalo: Breed, Lent & Co.

On Railway and other Injuries of the Nervous System. By John Eric Erichson, M. D., Fellow of the Royal College of Surgeons, Professor of Surgery and Clinical Surgery at the University College; Surgeon to the University College Hospital; Examiner in Surgery at tne University of London, and formerly so at the University of Durham and the Royal College of Physicians. Philadelphia: Henry C. Lea, 1867.

Essentials of the Principles ane Practice of Medicine, a Hand-book for Students and Practitioners. By Henry Hartshorne, M. D., Professor of Hygiene in the University of Pennsylvania, etc., etc. Philadelphia: Henry C. Lea, 1867.

The Principles and Practice of Disinfection. By Robert Bartholow, A. M., M.D., Professor of Materia Medica and Therapeutics in the Medical College of Ohio. Cincinnati: R. W. Carroll & Co., 1867.

Transactions of the Medical Society of the State of Kansas, for the year 1866.

Prize Essay on Medical and Vital Statistics, by Franklin B. Hough, M. D., of Lowville, N. Y., Superintendent of the New York State Census of 1855 & 1865.

Annual Report of the Commissioners of Emigration of the State of New York, for the year ending December 31, 1866.

Report of the Board of Health to the Common Council of the City of Troy, presented April 4, 1867.

MESSRS. A. SIMPSON & Co., New York, announce the early publication of the work of Prof. Julius Klob, of Vienna, on the Physiological Anatomy of the Female Sexual Organs, translated from the German by Drs. Kammerer and Dawson.

